# Long-term impact of intrauterine neuroinflammation and treatment with magnesium sulphate and betamethasone: Sex-specific differences in a preterm labor murine model

**DOI:** 10.1038/s41598-017-18197-x

**Published:** 2017-12-20

**Authors:** Andrew S. Thagard, Jessica L. Slack, Sarah M. Estrada, Avedis A. Kazanjian, Sem Chan, Irina Burd, Peter G. Napolitano, Nicholas Ieronimakis

**Affiliations:** 1 Department of OB/GYN, Division of Maternal Fetal Medicine, Madigan Army Medical Center, Tacoma, WA USA; 2 Department of Clinical Investigation, Madigan Army Medical Center, Tacoma, WA USA; 3 Division of Maternal Fetal Medicine, Johns Hopkins University, Baltimore, MD USA

## Abstract

Preterm infants are at significantly increased risk for lifelong neurodevelopmental disability with male offspring disproportionately affected. Corticosteroids (such as betamethasone) and magnesium sulphate (MgSO_4_) are administered to women in preterm labor to reduce neurologic morbidity. Despite widespread use of MgSO_4_ in clinical practice, its effects on adult offspring are not well known nor have sex-specific differences in therapeutic response been explored. The objective of our study was to examine the long-term effects of perinatal neuroinflammation and the effectiveness of prenatal MgSO_4_/betamethasone treatments between males and females in a murine model via histologic and expression analyses. Our results demonstrate that male but not female offspring exposed to intrauterine inflammation demonstrated impaired performance in neurodevelopmental testing in early life assessed via negative geotaxis, while those exposed to injury plus treatment fared better. Histologic analysis of adult male brains identified a significant reduction in hippocampal neural density in the injured group compared to controls. Evaluation of key neural markers via qRT-PCR demonstrated more profound differences in gene expression in adult males exposed to injury and treatment compared to female offspring, which largely showed resistance to injury. Prenatal treatment with MgSO_4_/betamethasone confers long-term benefits beyond cerebral palsy prevention with sex-specific differences in response.

## Introduction

Infants born preterm are at increased risk of neurologic injury resulting in neurodevelopmental disability, a global term encompassing varying degrees of neurologic impairment^1^. Damage to the central nervous system can occur through disruption of the delicate germinal matrix vasculature causing intraventricular hemorrhage (IVH) and as a result of hypoxia^2,3^. Long-term neurologic complications of prematurity include cerebral palsy^4^ and an increased frequency of attention-deficit/hyperactivity disorder^5^, among others.

Magnetic resonance imaging (MRI) studies suggest that children born preterm have decreased brain volumes compared to their term counterparts^6^, with the hippocampus – a region of the brain within the limbic system – appearing particularly vulnerable^7,8^. Prematurity in the context of a very low birth weight may impact an individual’s intelligence^9^. Human and animal studies demonstrate that the type and severity of neurologic injury associated with preterm labor varies between genders, with increased susceptibility in males^10–12^. Despite mounting evidence of an interplay between gender and the degree of perinatal brain injury, the underlying molecular and cellular mechanisms remain poorly understood.

There is no definitive intervention to prevent or treat prematurity-related neurodevelopmental disability, though antepartum administration of magnesium sulphate (MgSO_4_) has emerged as a mitigating strategy. Multiple randomized controlled trials summarized in meta-analyses^13,14^ suggest that administration of MgSO_4_ can reduce some neurologic complications of prematurity, most notably cerebral palsy. The estimated number needed to treat is 1 in 46^13^. The molecular pathway(s) by which MgSO_4_ confers neuroprotection in some but not all patients is unclear. Potential mechanisms of action include promoting hemodynamic stability, prevention of neural excitatory injury, and through antioxidant and/or anti-inflammatory effects^15^. While none of the major randomized controlled trials evaluating the benefit of MgSO_4_ for neuroprotection stratified results based on gender, sex steroids do influence neurogenesis^16^. Within the developing hippocampus in particular the interplay between estrogens and endocannaboid pathways leads to greater suppression of GABAergic inhibition in females compared to males^17^. If MgSO_4_ works in part by preventing neural injury from excitatory neurotransmitters, it is conceivable that a differential response based on sex could exist.

In addition to MgSO_4_, women at risk of delivering preterm receive a corticosteroid such as betamethasone (BMTZ in figures). The primary purpose of corticosteroids in preterm labor is to promote fetal lung maturation^18^, though they also reduce other prematurity-related morbidities including IVH^19^. Steroids have known anti-inflammatory properties which may be beneficial in inflammation-induced preterm labor. In the largest U.S. study evaluating the benefit of MgSO_4_ for neuroprotection^20^, 97 percent of patients received a corticosteroid (D. J. Rouse, MD, written communication, May 2012).

The use of animal models is critical to understanding the effects of neuroinflammation on brain development^21^ given limitations on human research. Studies using a murine model have provided insight into the molecular mechanisms of brain injury related to pre-term birth^12,22–24^. These studies have demonstrated the effects of *in utero* neuroinflammation including reductions in hippocampal volume that are detectable in adolescence and analysed sex-specific differences with notation of a greater degree of injury in male offspring^12^. Whether MgSO_4_ and corticosteroids reduce these negative outcomes in experimental adult animals and if there is a sex-specific difference in their response has not been tested.

**Figure 1:**
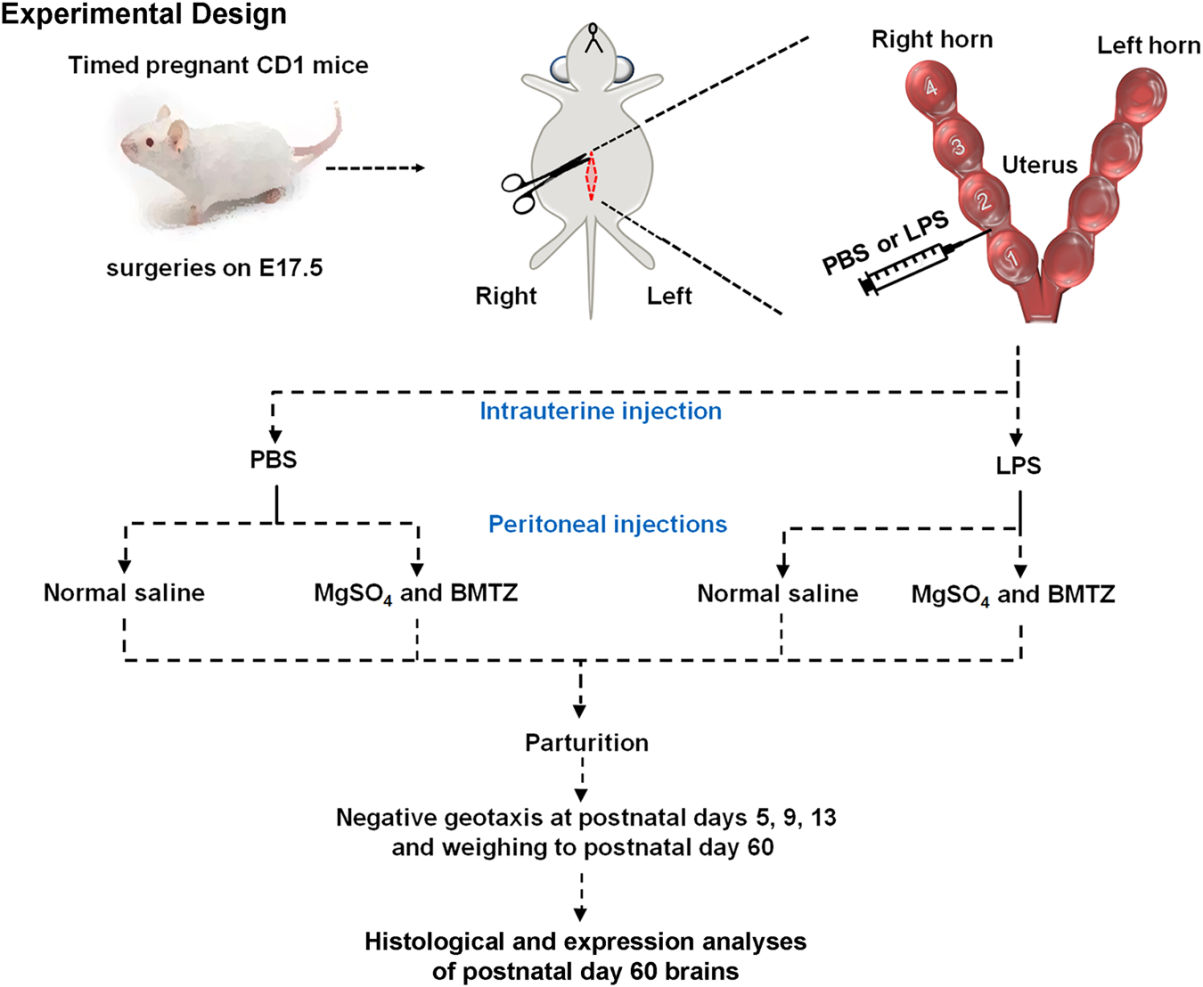
Schematic outlining the use of CD-1 mouse model of perinatal neuroinflammation resulting from the intrauterine injection of 50 μg of LPS vs. PBS controls on gestational age E17.5. Animals received either MgSO_4_ and betamethasone (BMTZ) or the vehicle (normal saline). Offspring were evaluated by negative geotaxis and weighed at the specified timepoints until euthanasia at postnatal day 60, at which point brains were collected for further analyses.

Based on the above, we hypothesize that prenatal MgSO_4_ and betamethasone will reduce the long-term impact of *in utero* neuroinflammation with male offspring benefitting to a greater degree than females. The objective of our study was to examine the long-term effects of perinatal neuroinflammation and the effectiveness of prenatal MgSO_4_ and betamethasone treatments between males and females in a murine model via histologic and expression analysis (Fig. 1).

## Results

### Survival outcomes

Approximately 600 offspring were delivered between the four groups (Fig. 1). All control dams that received phosphate buffered saline (PBS) delivered a litter with at least one surviving offspring. In contrast, dams that received lipopolysaccharide (LPS) exhibited significantly lower litter viability; 100% of litters survived with PBS and PBS + MgSO_4_/betamethasone vs. 63% with LPS and 75% with LPS + MgSO_4_/betamethasone (ANOVA P < 0.005). Mice randomized to receive LPS followed by treatment with MgSO_4_ and betamethasone had an increased litter survival rate compared to LPS plus placebo; however, this difference was not significant (student’s *t*-test P > 0.05). There was no difference in the number of offspring per litter assessed on post-natal day 5 with the exception of PBS vs. PBS + MgSO_4_/betamethasone control groups (student’s *t*-test P < 0.05).

### Neurodevelopmental testing and weights

**Figure 2:**
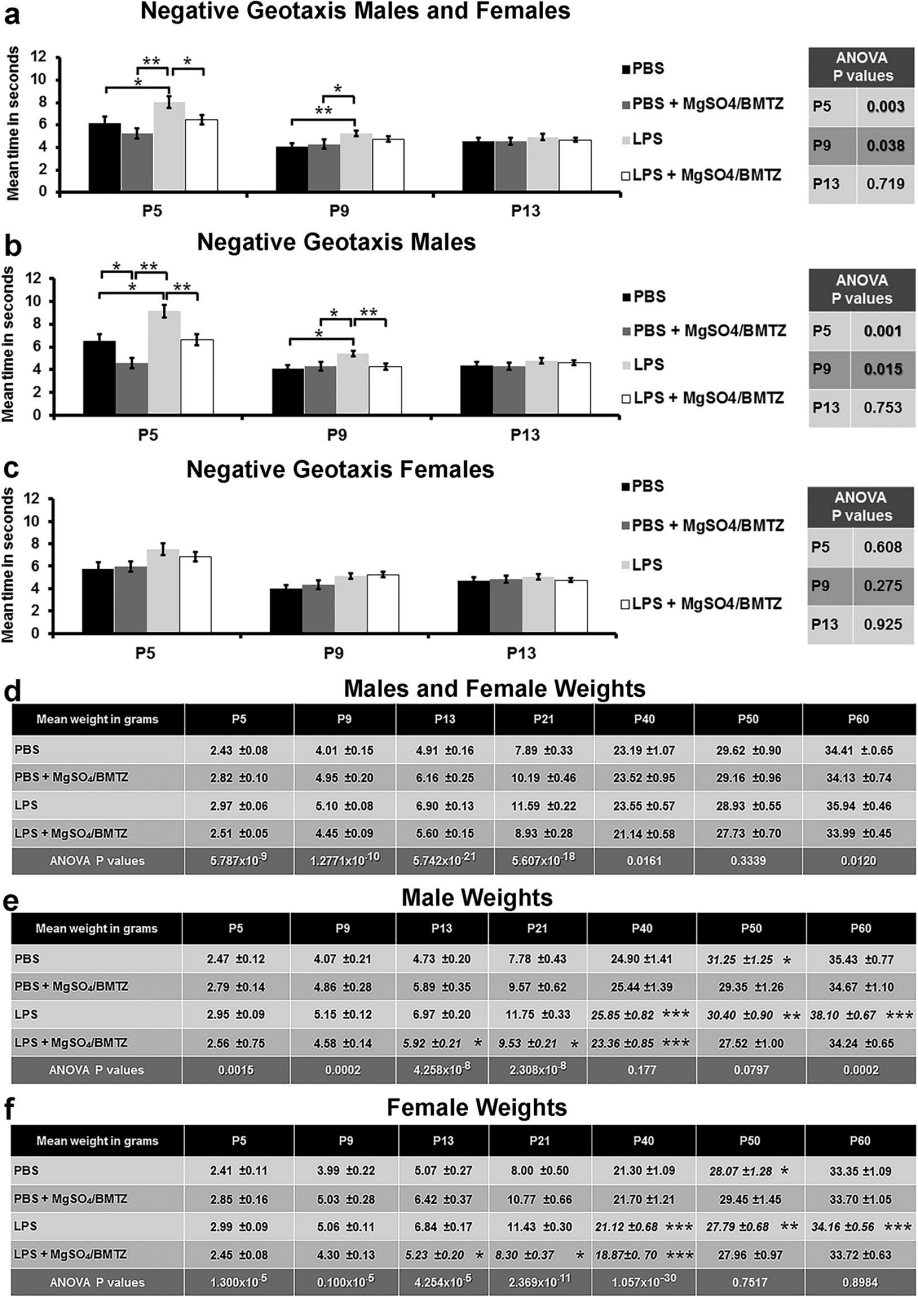
**(a)** Negative geotaxis conducted at postnatal days 5, 9, and 13. **(b** and **c)** Negative geotaxis stratified by sex from the same animals represented in Fig. 2a. (**d**,**e**) Weights from the same animals tested by negative geotaxis. For the negative geotaxis the following numbers were analyzed for each group per time point; PBS (n = 39 males, n = 38 females), PBS + MgSO_4_/BMTZ (n = 33 males, n = 33 females), LPS (n = 99 males, n = 108) LPS + MgSO_4_/BMTZ (n = 109, n = 97). *P < 0.05, **P < 0.005 by student’s *t*-test between experimental groups within each time point, there was no difference between males and females. **(d)** Weights monitored to postnatal day 60 for both female and male offspring. **(e** and **f)** Weights stratified by sex. *p < 0.05, **P < 0.005, ***P < 0.0005 denotes difference between male vs. female groups at respective time points by student’s *t*-test. For weights the same animals from Fig. 2a were measured through P5–13. Note by postnatal day 60 the number of animals was reduced to make weighing and post euthanasia analyses manageable; PBS (n = 22 males, n = 23 females), PBS + MgSO_4_/BMTZ (n = 22 males, n = 22 females), LPS (n = 66 males, n = 80 females) LPS + MgSO_4_/BMTZ (n = 69 males, n = 63 females). Animals within each group were randomly selected for reduction by blinded handlers that were not involved in the collection and analysis of the negative geotaxis or weight data. Error bars denote SEM.

Analysis of negative geotaxis testing in pre-weaned animals confirmed that offspring exposed to LPS demonstrated a delay in repositioning on postnatal days five (P5) and nine (P9) compared to PBS controls and those that prenatally received MgSO_4_/betamethasone (Fig. 2a). Stratifying these results by sex identified that only males were affected (Fig. 2b and c). By postnatal day 13 (P13), the impaired negative geotaxis response in males exposed to intrauterine LPS had normalized.

Mouse weights compared between treatment groups or when stratified by sex were significantly higher in both males and females on P5, P9, P13, P21, and P40. (Fig. 2d–f). On P50 neither male nor female groups were different. In contrast, male but not female groups were different on P60. A comparison between sexes indicated that LPS exposed males vs. females were significantly higher at P40, P50, and P60. LPS + MgSO_4_/betamethasone treated males vs. females weighed more on P13, P21, and P40, whereas PBS controls were only significantly different on P50.

### Histological and expression analyses

**Figure 3:**
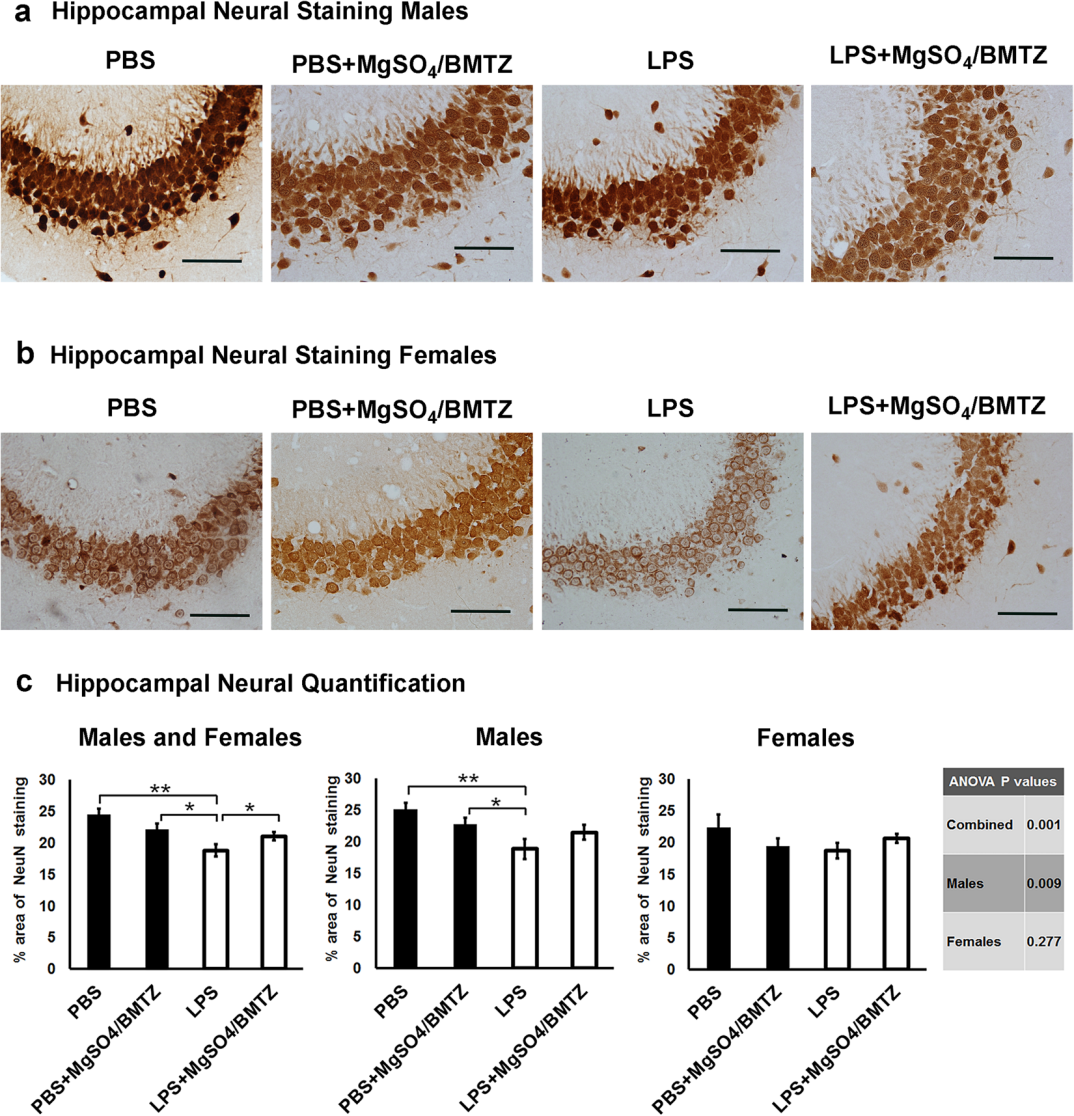
(**a** and **b**) Staining for NeuN with DAB was used to label neurons in coronal sections of male and female brains collected on postnatal day 60. High magnification photos of the hippocampus illustrate variations in neuron density between experimental groups. Scale bars = 100 µm. **(c)** Quantification of the neural density within the hippocampus (proportion of NeuN staining over the hippocampal area) was compared between experimental groups and sexes. Sections from n = 5 brains were analyzed per group for both males and females. *P < 0.05, **P < 0.005 by student’s *t*-test between experimental groups, there was no difference between males and females.

Hippocampal neural density, assessed via NeuN staining, was lower in the LPS group versus animals that received MgSO_4_/betamethasone and the PBS controls (Fig. 3). Once stratified by sex, this decline was attributed to the males alone, with a trend toward improvement with therapy following injury. In contrast, there was no difference in hippocampal neural density in female offspring among the four groups (Fig. 3c).

**Figure 4:**
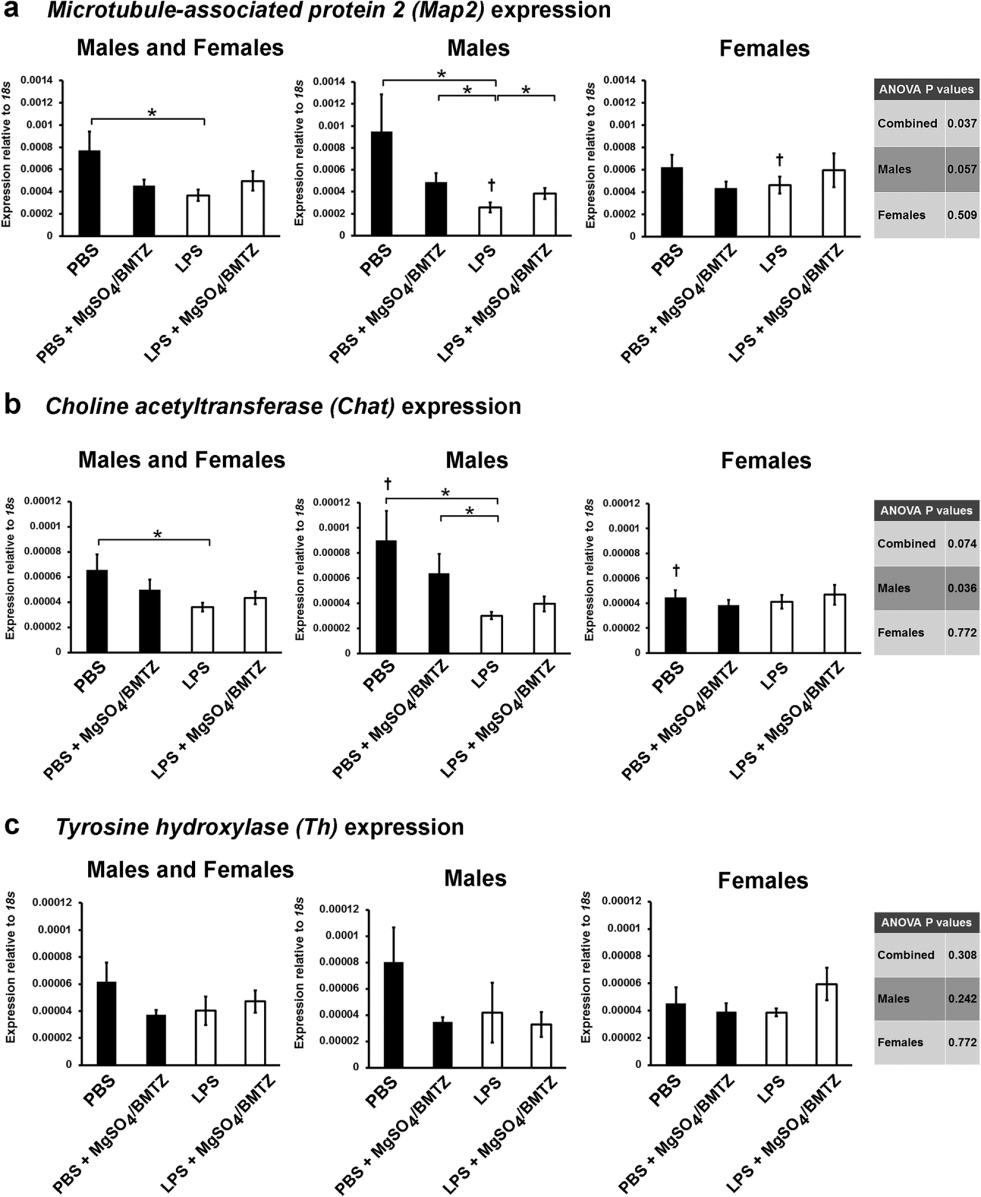
The expression of neuronal genes in whole brains (n = 8 male brains, n = 7 female brains) isolated from postnatal day 60 mice was analyzed by qRT-PCR. Expression levels were compared between experimental groups and by sex beginning with markers indicative of neurons and subtypes: **(a)** *Map2*, a gene expressed by most mature neurons. **(b)** *Chat*, expressed by motor neurons. **(c)** *Th*, expressed by dopaminergic neurons. *P < 0.05 by student’s *t*-test between experimental groups. ^†^Denotes P < 0.05 between sexes by student’s *t*-test for each respective experimental group. Error bars denote SEM.

Gene expression analysis revealed that males generally demonstrated more profound sequelae from neuroinflammation with some benefit conferred by MgSO_4_/betamethasone therapy. Although comparisons between all four groups were not always different by ANOVA, LPS vs. PBS males were generally significantly lower by student’s *t*-test. In contrast, expression of key neural targets in females appeared less affected by injury and consequently they did not derive significant benefit from therapy. Specifically, results indicate a decline in *Map2* in males exposed to LPS vs. those treated with MgSO_4_/betamethasone and the PBS controls (Fig. 4a). This decline was also significant between males and females exposed to LPS, but there was no difference among females when compared independently to males. Analogous to the expression pattern of *Map2*, *Chat* was significantly lower with LPS exposure between male groups (Fig. 4b). However, there was no rescue of *Chat* expression with LPS + MgSO_4_/betamethasone treated males, nor was there a difference between male and female LPS exposed groups. Females also showed little difference between groups, yet the PBS group was significantly lower for *Chat* expression vs. PBS males. Contrary to these results, *Th* expression varied but was not different between the sexes or treatment groups (Fig. 4c).

**Figure 5:**
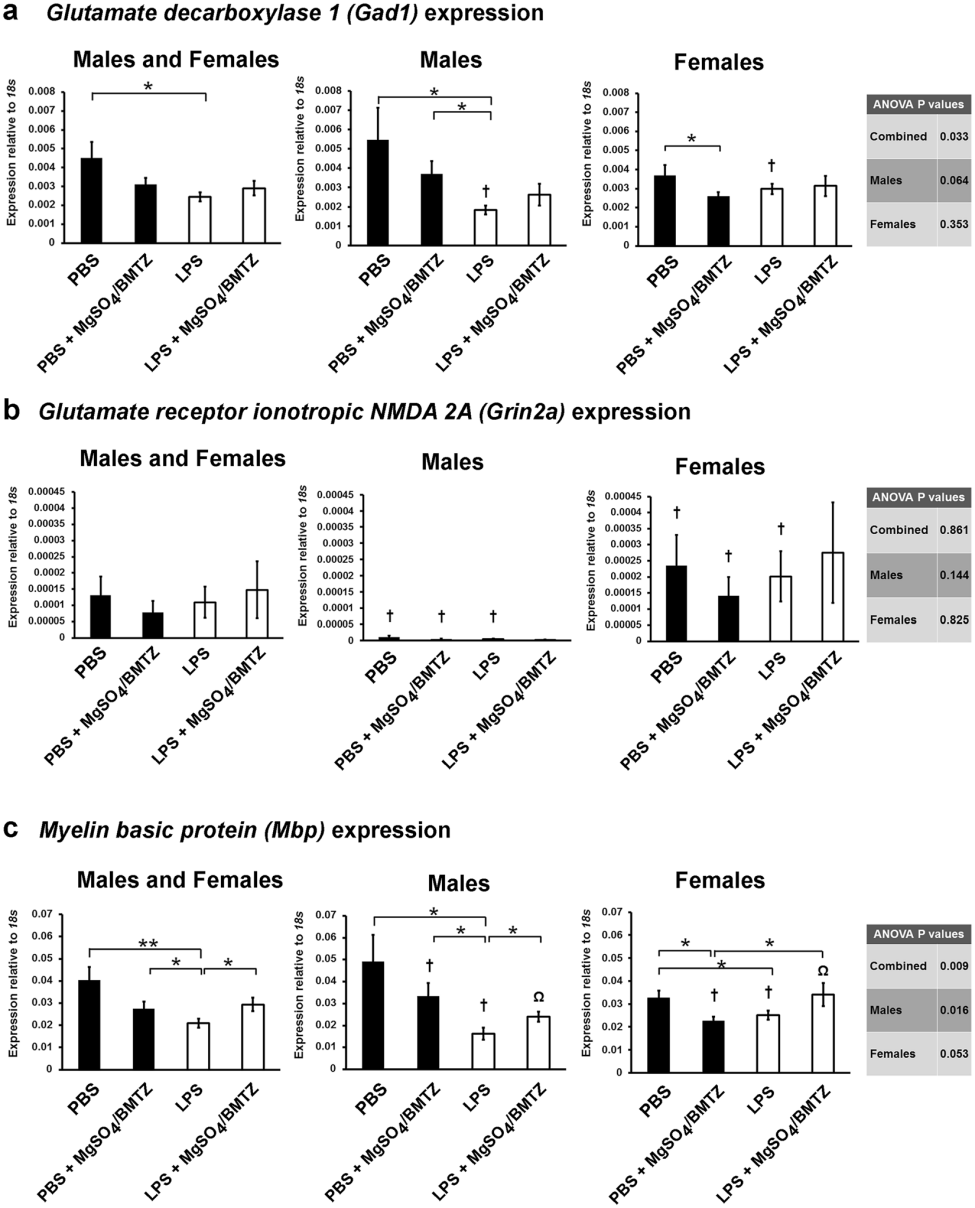
The expression of genes in whole brains (n = 8 male brains, n = 7 female brains) by qRT-PCR, related to neurotransmission: **(a)** *Gad1*, an enzyme essential for the conversion of glutamate to gamma-aminobutyric acid (GABA). **(b)** *Grin2a*, the alpha subunit of the glutamate receptor NMDA 2 subunit. **(c)** *Mbp*, an essential component of myelin sheaths and in turn neurotransmission. *P < 0.05 by student’s *t*-test between experimental groups. Ω P = 0.05 and ^†^P < 0.05 between sexes by student’s *t*-test for each respective experimental group. Error bars denote SEM.

The expression of the neurotransmission related gene *Gad1* was significantly lower in LPS exposed males but not different with MgSO_4_/betamethasone treatment. The LPS male group also had reduced expression in comparison to LPS females (Fig. 5a). The only difference in *Gad1* expression for females was between the PBS vs. PBS + MgSO_4_/betamethasone control groups. In comparison to *Gad1*, the expression for *Grin2a* was not different between treatment groups (Fig. 5b). There was, however, a significant difference between male vs. females groups, with the exception of LPS + MgSO_4_/betamethasone treated mice.

Evaluation of *Meylin basic protein* (*Mbp*), the second most abundant protein in the brain and an essential component of myelination^25^, revealed reduced expression with LPS mediated injury in both males and females (Fig. 5c). Treatment with MgSO_4_/betamethasone following injury significantly rescued *Mbp* expression but only in males. Similar to *Gad1*, MgSO_4_/betamethasone treatment significantly reduced the expression of *Mbp* in PBS female controls. There were also significant differences in *Mbp* expression between males vs. females groups, with the exception of PBS treated mice.

**Figure 6:**
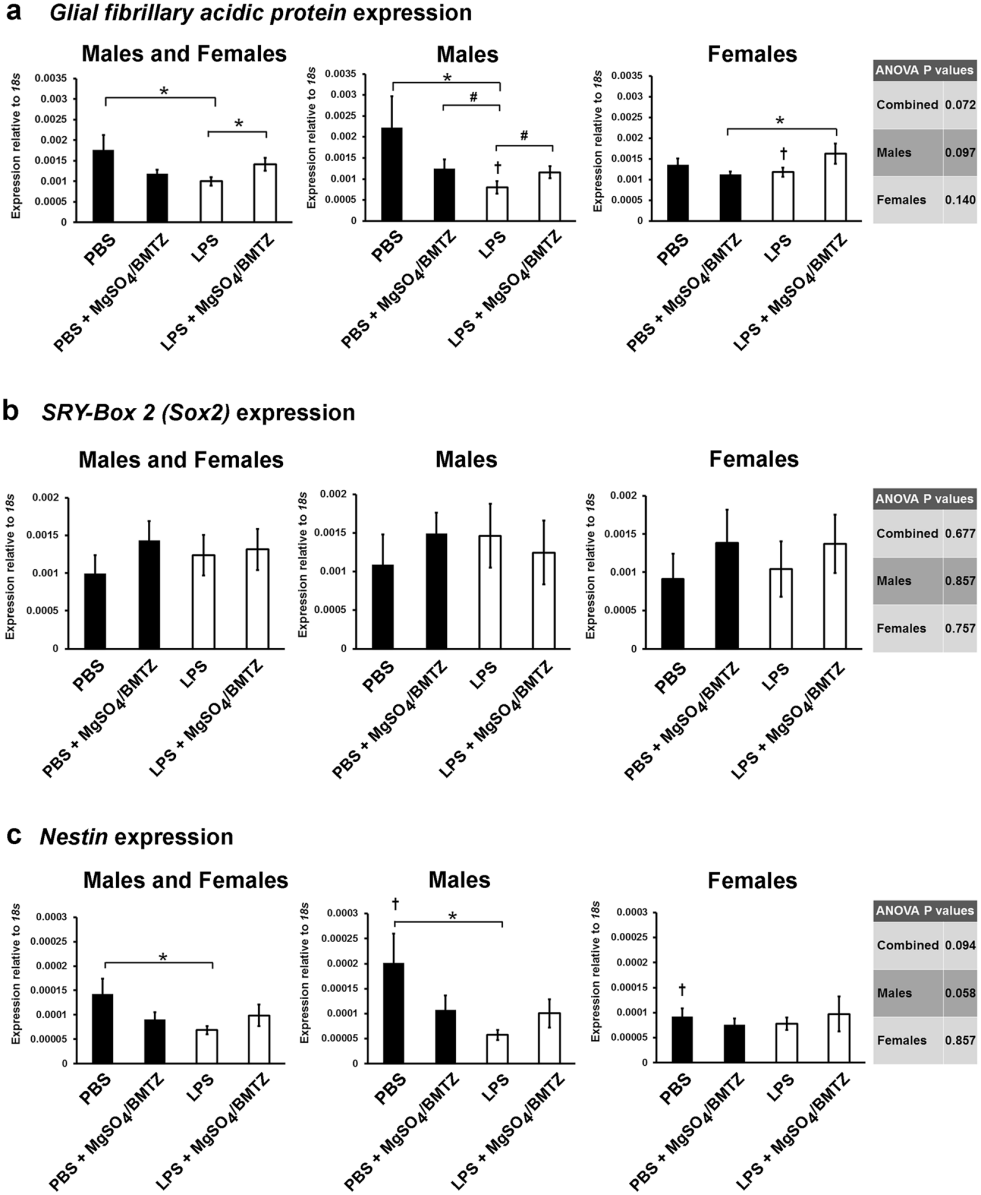
The expression of genes in whole brains (n = 8 male brains, n = 7 female brains) by qRT-PCR, related to glia, neural progenitors and stem cells: **(a)** *Gfap*, a protein expressed by the majority of glia. **(b)** *Sox2*, a marker of neural stem cells and progenitors. **(c)** *Nestin*, a gene that defines and is required by neural stem cells. ^#^P = 0.05 and *P < 0.05 by student’s *t*-test between experimental groups. ^†^P < 0.05 between sexes by student’s *t*-test for each respective experimental group. Error bars denote SEM.

*Glial fibrillary acidic protein* (*Gfap*) was assayed to evaluate astrocytes. Results indicate a significant decline in *Gfap* in male brains between PBS controls and LPS exposed animals (Fig. 6a). Treatment with MgSO_4_/betamethasone in LPS exposed males resulted in a higher expression but this was not significantly different compared to LPS alone. Males exposed to LPS also had significantly lower expression than females. Females groups were similar with the exception of those that received MgSO_4_/betamethasone and either PBS or LPS. In contrast, the expression of *Sox2* was not different between groups or sexes (Fig. 6b). Finally, *Nestin* expression was significantly different between PBS vs. LPS treated males and between PBS males vs. PBS females (Fig. 6c).

## Discussion

While MgSO_4_ is described as a means of providing global neuroprotection, research to date has almost exclusively focused on a reduction in the incidence of cerebral palsy in the first few years of life. The intent of this study was to explore the long-term effects of neuroinflammation and the benefits of MgSO_4_ and betamethasone therapy extending into adulthood and to determine if there is a sex-specific difference in injury and response.

To test our hypothesis, we modified an established mouse model of preterm labor and perinatal brain injury which exposed offspring to insult in the form of LPS i*n utero*. A subset of animals was allocated to receive MgSO_4_ and betamethasone therapies 30 minutes after injury. Body righting mechanisms, strength, and coordination – skills modulated in part by the hippocampus – were assessed in juvenile offspring using negative geotaxis testing. Brains from adult animals were processed either for histology or expression analysis of key neural markers, including those indicative of neurons, transmission, glia, white matter, and stem cells.

**Figure 7:**
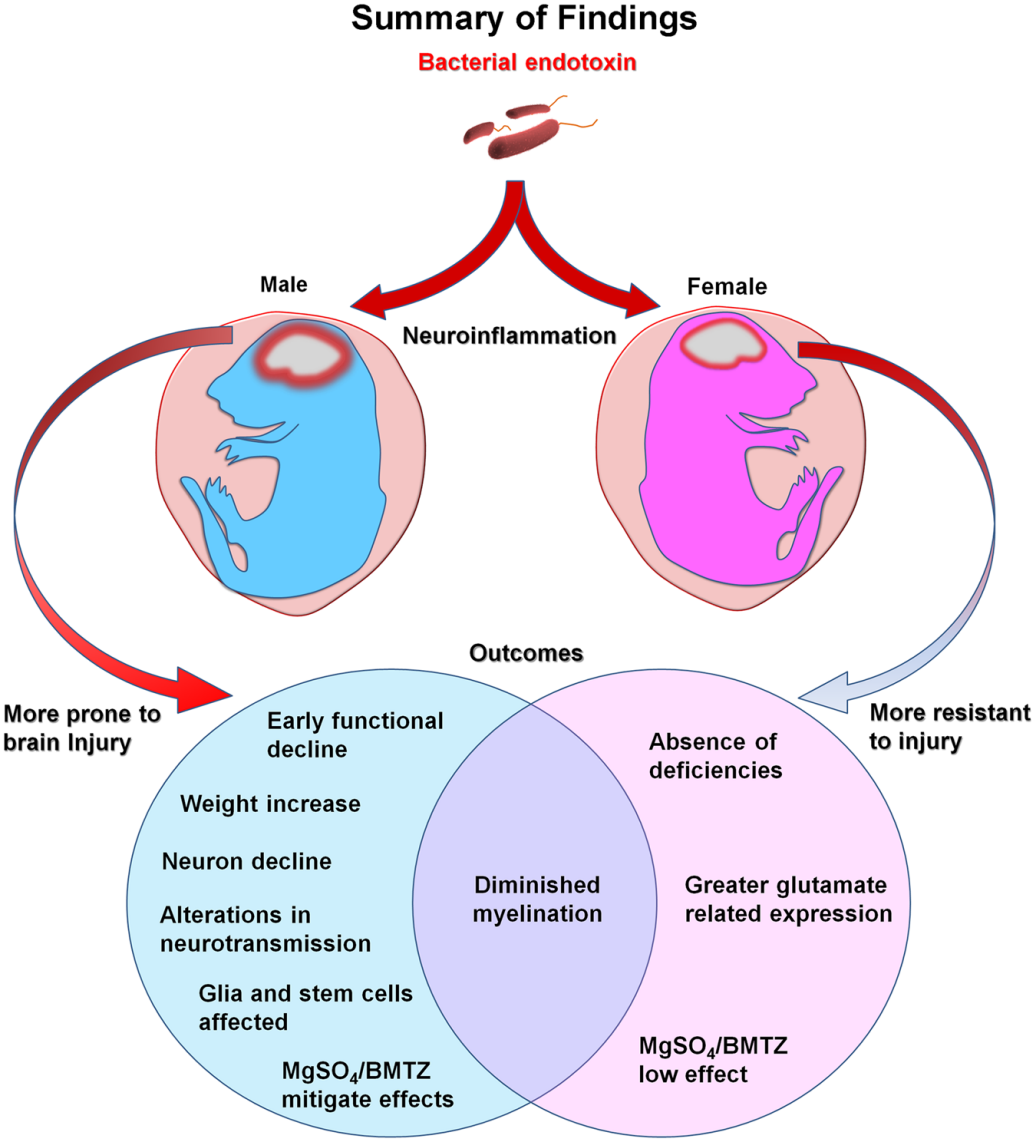
Summarizing of our findings related to sex differences with the long term effects of perinatal neuroinflammation. The majority of deficiencies related to LPS treatment were detected in males, suggesting that females are resistant to neuroinflammatory injury. Sex differences were associated with greater expression of glutamate related genes and less effectiveness of MgSO_4_/BMTZ treatment in females, likely due to the absence of injury.

Our results suggest that exposure to intrauterine inflammation impacts male and female offspring differently (Fig. 7). Early in life, males exposed to LPS demonstrated impaired performance of negative geotaxis testing with improvement in those treated with MgSO_4_/betamethasone (Fig. 2b). The normalization of geotaxis by P13 in males may be attributed to developmental plasticity or a decline in the sensitivity of this test due to the increasing awareness of the animals as they opened their eyes and became more mobile with advancing age. In contrast, female offspring exhibited no differences between groups. Interestingly, both males and female exposed *in utero* to LPS tended to weight more than PBS controls in early life. Though weights fluctuated into adolescence, males exposed to LPS were larger at the time of euthanasia on P60. Considering that no differences in litter sizes were observed, the changes in weight cannot be attributed to the attrition of siblings *in utero* (Fig. 1c). It is possible that weight increases with LPS are either a unique facet of mouse biology or related to systemic effects that influence nutrient uptake^26^.

In adult offspring, both sexes demonstrated a decline in a key marker of myelination (*Mbp*); however, interrogation of targets linked to neurotransmission, glia, and stem cells generally revealed more profound injury in males with females largely unaffected. Similarly, histologic analysis of hippocampal neural density via NeuN staining identified a decline in male but not female offspring following injury (Fig. 3). Prenatal treatment with MgSO_4_/betamethasone rescued expression of some (*Map2*, *Mbp*) but not all genes (*Chat*, *Gad1*, *Nestin*). Largely resistant to neuroinflammation, female offspring appeared to derive little benefit from MgSO_4_/betamethasone therapy. Of note, females expressed greater levels of *Grin2a* which encodes the NMDA receptor 2 alpha subunit (NR2A) (Fig. 5b). NR2A is a component of NMDA receptor channels and is expressed at lower levels in the embryonic brain^27^ but gradually replaces the NR2B subunit during postnatal development. Taking into consideration the role of NMDA receptors and their sensitivity to MgSO_4_^28^, it is possible that sex differences in the composition of NMDA receptors or other components of glutamate signaling (e.g. Gad1), may influence perinatal brain injury^29^. However, it is unclear from our study whether the differential response to therapy is due to sex-specific mechanisms related to glutamate signaling or other molecular factors.

Sex-specific differences were also noted for *Chat* between PBS controls (Fig. 4b). Chat which synthesizes the neurotransmitter acetylcholine has been implicated in neurodegenerative and behavioral diseases such as Alzheimer’s and schizophrenia^30–32^. The activity of Chat and the relative abundance of *Chat* mRNA can be modulated by sex-hormones^33,34^. Treatment with estrogen can elevate the expression of *Chat* in female but not male rodents^33,35^. In contrast, administration of testosterone does not affect the number of Chat positive neural cells, though following gonadectomy males do show a reduction^36,37^. Our results suggest a baseline sex-specific difference in *Chat* expression that reduced with LPS only in males (Fig. 4b). Though our study does not provide a mechanism for this difference, we theorize that female sex-specific hormones infer protection from perinatal neuroinflammation and therefore the expression of *Chat* is preserved. In turn, males may lack this defense and be prone to perinatal brain injury that alters the expression of key neural genes into adulthood.

In our multiple comparisons of gene expression, the possibility of a type I error may have contributed to some of the sex-specific differences observed. Though corrections (such as Bonferroni) are used to reduce type I error, these approaches are more commonly employed for high-throughput gene analysis such as microarrays. The probability of false discovery is attributed in part to the sensitivity of such screening assays and the large number of genes analyzed. Our analysis relied on specific primers to examine a small number of pre-selected genes, thereby limiting the possibility of a type I error^38^. Though type I error may still arise with multiple comparisons such as those presented in our study, we elected to omit the correction in order to avoid type II error. Nevertheless, these findings warrant further examination to support the validity of neurologic sex-specific differences.

The increased morbidity and mortality of male offspring in the context of prematurity is well documented^39^. In particular, male infants born preterm are at increased risk for neurodevelopmental disability including cognitive deficits, language impairment, and cerebral palsy, among others^40,41^. The mechanism conferring increased susceptibility in preterm male offspring remains unknown though differences in intrauterine adaptations, rates of maturation, and fetal sex steroids have been proposed^42^. Further research into sex-specific responses to inflammation-induced neurologic injury may yield insight into specific vulnerabilities of the male fetus and allow more targeted interventions. Additionally, stratifying existing data from the major randomized controlled trials could determine if a sex-based difference in response to MgSO_4_ therapy exists in humans.

The main limitation of this study is its reliance on an animal model. While we have selected one that uses an inflammation-based form of preterm labor, no model can completely reflect the complexities of human preterm labor. Rodents, including mice, are born at a more neurologically immature state than their human counterparts and brain development and growth continues into the postnatal period^43^. Thus, the impact of prenatal injury may be different in humans compared to mice. Furthermore, our study examines the benefits of MgSO_4_/betamethasone in brain injury resulting from endotoxin exposure not active bacterial infections. A comparison of MgSO_4_/betamethasone in models that rely on bacterial inoculation such as Group B S*treptococcus* ^44^ and *Escherichia coli* ^45,46^, would be informative in assessing sex-specific differences between pathogens commonly associated with preterm labor.

A second limitation is that our experimental design simulates the current clinical regimen for MgSO_4_/betamethasone but does not address if the effectiveness of these therapies varies with respect to dosing and timing of administration. It is conceivable that administering MgSO_4_ therapy prior to preterm labor may provide additional benefit, as indicated in trials for preenclampsia^47,48^. However, this agent is a “high risk” medication that currently requires hospitalization and a continuous intravenous infusion. Our results suggest that in the absence of neuroinflammation, MgSO_4_/betamethasone may negatively alter normal gene expression levels as evident in female controls with *Gad1* and *Mbp* expression. Therefore, without biomarkers to predict preterm deliveries, the dosing and safety of these treatments would have to be carefully evaluated for prophylactic use.

Our study has several strengths. We selected the model of preterm labor that is believed to most accurately reflect the most common form of preterm labor in humans rather than those that employ progesterone withdrawal or overt infection. Additionally, we carefully selected the timing and dosage of injury and therapeutic interventions based on prior published studies and worked to establish a phenotype of injury to correlate with histological and qRT-PCR findings.

In a murine model, our research demonstrates that exposure to intrauterine inflammation produces a differential response based on sex, with male offspring more profoundly affected. Prenatal administration of MgSO_4_/betamethasone partially ameliorated this damage but significant long-term benefit was only observed in male offspring. Our findings suggest that MgSO_4_/betamethasone confer long-term benefits beyond cerebral palsy prevention with sex-specific differences in response. Our study supports further research into sex-responses to perinatal neuroinflammation and development of precision medicine approaches that address the effectiveness of MgSO_4_/betamethasone treatments between male and female offspring.

## Methods

This protocol was approved by the Madigan Army Medical Center Institutional Animal Care and Use Committee (MAMC IACUC). Animals involved in this study were maintained in accordance with the ‘Guide for the Care and Use of Laboratory Animals’ published by the National Research Council/Institute of Laboratory Animal Research (ILAR). We modified an existing model of inflammation-induced preterm labor and perinatal brain injury developed by Elovtiz *et al*. in 2003^12,21–24,49–51^. CD-1 timed pregnant mice were shipped from the vendor (Harlan Laboratories, Indianapolis IN) and arrived at our facility for a period of acclimation. On day 17.5 of a 19–21 day gestation, 88 dams were placed under general anesthesia and the bicorunate uterus was accessed by laparotomy (Fig. 1a).

Animals were randomized to receive an intrauterine injection of 50 µg LPS derived from *Escherichia coli* (Sigma–Aldrich, St. Louis, MO) or an equivalent volume (100 μl) of vehicle (PBS)^22^. Thirty-four animals were assigned to each LPS group compared to 10 animals in the PBS control groups based on research indicating that 37 percent of dams exposed to this dose of LPS will deliver a surviving litter^12^.

Thirty minutes after surgery, dams were further assigned to receive a combination of betamethasone (0.1 mg subcutaneous injection) and MgSO_4_ (270 mg/kg loading dose followed by 27 mg/kg every 20 minutes for four hours injected subcutaneously)^52,53, all at 100 μl per injection^. We selected this interval based on research demonstrating an increase in inflammatory serum cytokines that is measurable 30 minutes following LPS administration^54^. Dams remained in single cage housing and were closely monitored under the supervision of an attending veterinarian until delivery.

Surviving litters were reared until postnatal day 60. Weaning took place on postnatal days 20–22. Once sex could reliably be determined, animal numbers were randomly reduced from up to 16 to six animals per litter with an equal number of male and female offspring. Euthanasia of pups was accomplished with a sodium pentobarbital-based compound (Fatal-Plus, Vortech Pharmaceuticals, Dearborn MI), adolescents and adults underwent cervical dislocation under general anesthesia.

Neurodevelopmental assessment in the form of negative geotaxis testing was performed on postnatal days 5, 9, and 13 to assess body righting mechanisms, strength, and coordination – deficiencies that may result from exposure to perinatal neuroinflammation^55^. For this test, the pup was placed head down on a screen angled 45 degrees and the time in seconds for the animal to convert to a heads up orientation was measured^12,56^. Animals acclimated to the testing room for at least 60 minutes before assessments. Testing was conducted at the same time each session and by the same core group of investigators. Animals were weighed prior to testing and individuals were tracked using tail markings. A subset of tests were filmed and audited by a separate investigator to ensure consistency in test performance and grading.

On postnatal day 60, any remaining offspring were euthanized. Whole brains were collected for either histological or transcriptional analysis. Brains destined for histological analysis were fixed in neutral buffered formalin and embedded in paraffin. Fixed brains were subsequently sectioned 8 µM thick and stained for NeuN, a marker of mature neurons^57^. NeuN staining was conducted following antigen retrieval with sodium citrate buffer pH 6.0 + 0.05% Tween 20 for 30 minutes at 95 °C and blocking with 2.5% horse serum for 30 minutes. Subsequently, the monoclonal rabbit anti-NeuN (Abcam, Eugene, OR [catalog # ab17748]) was diluted at 1:2000 in PBS +1% BSA and applied overnight at 4 °C. The next day, the staining was visualized using the ImmPRESS anti-rabbit peroxidase kit and ImmPACT DAB substrate (both from Vector Labs, Burlingame, CA) in accordance with the manufacturer’s protocols. Hippocampal neural density was calculated from photographs of NeuN stainings in ImageJ (NIH, Bethesda, MD) by analyzing the percentage of DAB (NeuN) staining within the hippocampal cross-sectional area^58^.

For expression analysis, brains were flash frozen in liquid nitrogen and homogenized for total RNA isolation using the RNeasey Lipid Tissue Mini kit (Qiagen, Hilden, Germany). First stand cDNA was generated using the GoScript Reverse Transcriptase System (Promega, Madison, WI) for quantitative reverse-transcription PCR (qRT-PCR) with the FastStart SYBR Green master mix (Roche, Basel, Switzerland)). PCR reactions were run for 40 cycles using a Roche 480 II lightcycler. The relative expression of each target was calculated by normalizing to ribosomal *18s* by the ΔCt method. All primers were generated by the Harvard PrimerBank, with the exception of *18s* rRNA^59,60^. Primer sequences are listed in Supplemental Table 1.

Genes associated with neurons, neural transmission, glia, white matter, and stem cells were targeted for expression analysis. All genes tested as part of this study are reported as follows; *Microtubule associated protein 2 (Map2*) which encodes a cytoskeletal protein indicative of neurons^61^, was selected as a general neural marker. *Choline acetyltransferase* (*Chat*), expressed by cholinergic neurons, and *Tyrosine hydroxylase* (*Th*), essential for catecholaminergic processes^62,63^, were examined as markers of neurotransmission. Glutamate and myelination, two related processes that are connected with perinatal brain injury^64^ were also compared between groups using the targets Glutamate decarboxylase 67 kDa (Gad67) encoded by *Gad1* ^65^, *Glutamate receptor ionotropic NMDA 2AGrin2a*)^25,66^, and *Meylin basic protein* (*Mbp)* ^25^. To assess astrocytes, the expression of *glial fibrillary acidic protein* (*Gfap*) was assayed^67^. Finally, neural progenitors and stem cells were evaluated by the expression of *SRY-box 2* (*Sox2*) and *Nestin* ^68^.

For all experiments, statistical significance was determined by student’s *t*-test or one-way ANOVA. P values < 0.05 was deemed significant. In these statistical analyses, multiple comparisons were not corrected to circumvent type II error, though type I error - false discovery may arise. The datasets generated during and/or analyzed during the current study are available from the corresponding author on request.

## Electronic supplementary material

Supplemental Table 1.
