# Supplementary material for: Long-term impact of intrauterine neuroinflammation and treatment with magnesium sulphate and betamethasone: Sex-specific differences in a preterm labor murine model
Source: Sci Rep. 2017 Dec 20;7:17883. doi: 10.1038/s41598-017-18197-x (PMC5738437; doi:10.1038/s41598-017-18197-x)
Supplement: Supplementary file 1 — Supplemental Table 1. [file 41598_2017_18197_MOESM1_ESM.pdf]

Long-term impact of intrauterine neuroinflammation and treatment with magnesium sulphate and betamethasone: Sex-specific differences in a preterm labor murine model

Andrew S. Thagard, MD[1], Jessica L. Slack, PhD[2], Sarah M. Estrada, MD[1], Avedis A. Kazanjian, PhD[2], Sem Chan, BS[2] , Irina Burd, MD, PhD[3], Peter G. Napolitano, MD[1], Nicholas Ieronimakis, PhD[2*]

[1]Department of OB/GYN, Division of Maternal Fetal Medicine, Madigan Army Medical Center, Tacoma, WA.
[2]Department of Clinical Investigation, Madigan Army Medical Center, Tacoma, WA.
[3] Division of Maternal Fetal Medicine, Johns Hopkins University, Baltimore MD.

Supplemental Table 1: Primer sequences used for qRT-PCR

Gene	Forward	Reverse	Product size base pairs
18s rRNA	TTGACGGAAGGGCACCACCAG	GCACCACCACCCACGGAATCG	130
Map2	AAGAAACAGCTAATCTGCCACC	GGCTCTTGCTTATTCCATCAGTG	153
Chat	GGCCATTGTGAAGCGGTTTG	GCCAGGCGGTTGTTTAGATACA	138
Th	TCTCCTTGAGGGGTACAAAACC	ACCTCGAAGCGCACAAAGT	151
Gad1	CACAGGTCACCCTCGATTTTT	ACCATCCAACGATCTCTCTCATC	176
Nmdar2a	ACGTGACAGAACGCGAACTT	TCAGTGCGGTTCATCAATAACG	100
Mbp	TCACAGCGATCCAAGTACCTG	CCCCTGTCACCGCTAAAGAA	125
Gfap	ACCAGCTTACGGCCAACAG	CCAGCGATTCAACCTTTCTCT	198
Sox2	GCGGAGTGGAAACTTTTGTCC	CGGGAAGCGTGTACTTATCCTT	157
Nestin	CCCACCTATGTCTGAGGCTC	GGGCTAAGGAGGTTGGATCAT	212
